# Human umbilical cord mesenchymal stem cell transfusion in immune non-responders with AIDS: a multicenter randomized controlled trial

**DOI:** 10.1038/s41392-021-00607-2

**Published:** 2021-06-09

**Authors:** Lifeng Wang, Zheng Zhang, Ruonan Xu, Xicheng Wang, Zhanjun Shu, Xiejie Chen, Siyu Wang, Jiaye Liu, Yuanyuan Li, Li Wang, Mi Zhang, Wei Yang, Ying Wang, Huihuang Huang, Bo Tu, Zhiwei Liang, Linghua Li, Jingxin Li, Yuying Hou, Ming Shi, Fu-Sheng Wang

**Affiliations:** 1 Treatment and Research Center for Infectious Diseases, Fifth Medical Center of Chinese PLA General Hospital, Beijing, China; 2 Institute of Hepatology, National Clinical Research Center for Infectious Disease, Shenzhen Third People’s Hospital, The Second Affiliated Hospital, School of Medicine, Southern University of Science and Technology, Shenzhen, Guangdong Province China; 3 Yunnan Provincial Hospital of Infectious Diseases, Kunming, China; 4 Xinjiang Uygur Autonomous Regional the Sixth People’s Hospital, Urumqi, China; 5 Guangzhou Eighth People’s Hospital, Guangzhou Medical University, Guangzhou, China; 6 NHC Key Laboratory of Enteric Pathogenic Microbiology, Jiangsu Provincial Center for Disease Control and Prevention, Nanjing, China

**Keywords:** Clinical trials, Clinical trial design

## Abstract

We examined the safety and efficacy of human umbilical cord mesenchymal stem cell (hUC-MSC) infusion for immune non-responder (INR) patients with chronic HIV-1 infection, who represent an unmet medical need even in the era of efficient antiretroviral therapy (ART). Seventy-two INR patients with HIV were enrolled in this phase II randomized, double-blinded, multicenter, placebo-controlled, dose-determination trial (NCT01213186) from May 2013 to March 2016. They were assigned to receive high-dose (1.5 × 10^6^/kg body weight) or low-dose (0.5 × 10^6^/kg body weight) hUC-MSC, or placebo. Their clinical and immunological parameters were monitored during the 96-week follow-up study. We found that hUC-MSC treatment was safe and well-tolerated. Compared with baseline, there was a statistical increase in CD4+ T counts in the high-dose (*P* < 0.001) and low-dose (*P* < 0.001) groups after 48-week treatment, but no change was observed in the control group. Kaplan–Meier analysis revealed a higher cumulative probability of achieving an immunological response in the low-dose group compared with the control group (95.8% vs. 70.8%, *P* = 0.004). However, no significant changes in CD4/CD8+ T counts and CD4/CD8 ratios were observed among the three groups. In summary, hUC-MSC treatment is safe. However, the therapeutic efficacy of hUC-MSC treatment to improve the immune reconstitution in INR patients still needs to be further investigated in a large cohort study.

## Introduction

Despite effective control of HIV replication with combined antiretroviral therapy (ART), around 10–40% of treated patients with HIV-1 fail to achieve normalization of CD4+ T counts and are referred to as “inadequate immunological responders,” “immunodiscordant responders,” or “immune non-responders” (INRs).^1,2^ Such patients remain at greater risk of morbidity and mortality than immune responders.^3,4^ The possible mechanisms of INR include decreased bone marrow hematopoiesis, insufficient thymic output, residual virus replication, and aberrant immune activation, which eventually lead to reduced CD4 production and excessive CD4 destruction.^5,6^ A modified regimen of ART, ART intensification, immunomodulators (e.g., vitamin D, interleukin-2, interleukin-7), immunosuppressive agents, and probiotics have been used to promote the immune restoration of CD4 for INR patients with HIV-1, but did not achieve satisfactory clinical benefits.^7–13^

Mesenchymal stem cells (MSCs) are adult stem cells and can originate from the bone marrow, adipose tissue, placenta, umbilical cord, cord blood, peripheral blood, and the liver. MSCs have considerable therapeutic effects due to their migration, differentiation, immune-modulation, and regeneration abilities. Numerous published studies have reported the successful use of MSCs for treating different diseases, such as acute severe graft-versus-host disease (GVHD), heart failure, liver injury, osteoarthritis, autoimmune diseases, and liver cirrhosis. Previously, we found that hUC-MSC transfusion is safe and can decrease systemic immune overactivation, increase circulating naïve and central memory CD4 T cell counts, and restore HIV-1 specific interferon (IFN)-γ and IL-2 production in INRs.^14^ However, only seven patients were included and there was no dose escalation. Recently, Trujillo-Rodríguez et al.^15^ treated INR patients using human allogeneic mesenchymal stromal cells from adipose tissue infusions, and reported no effectiveness for either improving immune recovery or for reducing immune activation or inflammation in those patients. However, only five patients were enrolled.

Therefore, we designed this multicenter, randomized, double-blinded, placebo-controlled, dose determination phase II clinical trial of hUC-MSC transfusion for INR patients with HIV-1. The primary endpoints were to evaluate the immune restoration of CD4 counts after hUC-MSC transfusion. The secondary endpoint was to examine the changes in CD8 counts and CD4/CD8 ratio, and confirm the safety of hUC-MSC transfusion.

## Results

**Fig. 1:**
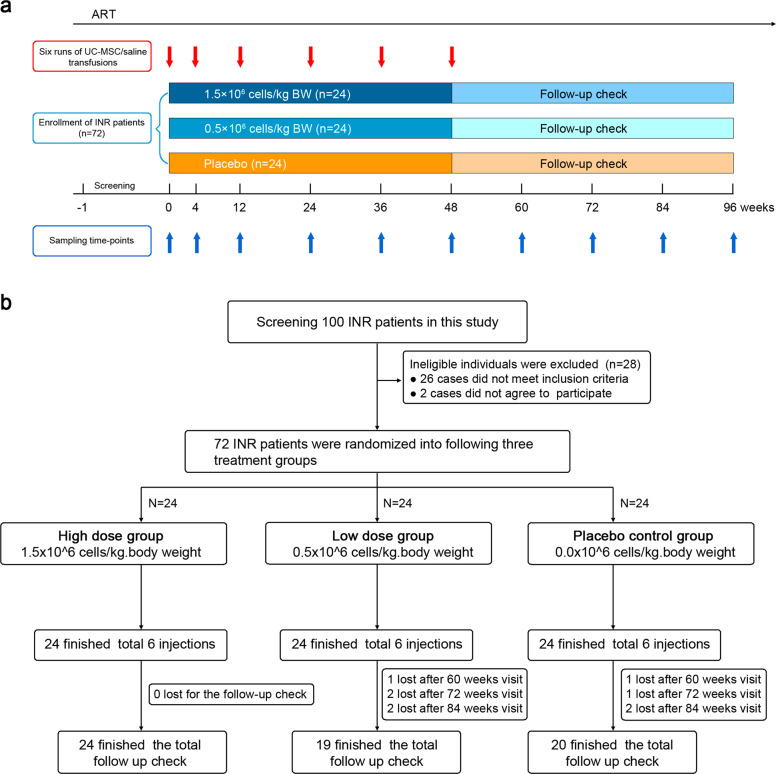
Study design. **a** Graphic overview of the study schedule; and **b** CONSORT (Consolidated Standards of Reporting Trials) diagram

Of 100 recruited patients, 26 did not meet the inclusion criteria after the baseline screening, and two patients declined to continue the study before starting the trial. A total of 72 patients were enrolled in this trial and were randomly assigned to the high-dose (1.5 × 10^6^ cells/kg body weight hUC-MSC transfusion, 24 patients), low-dose (0.5 × 10^6^ cells/kg body weight hUC-MSC transfusion, 24 patients), or control (saline, 24 patients) group, and were treated on months 0, 1, 3, 6, 9, and 12. Five patients in the low-dose group and four patients in the placebo group were lost after 60 weeks’ treatment, while the remaining patients finished the 96-week follow-up (Fig. 1a). The baseline characteristics of the total 72 participants are summarized in Table 1. All patients enrolled in the present study did not participate in phase I treatment of MSC as previously reported.^14^ The clinical variables, including age, sex, weight, BMI (body mass index), HIV infection route, diagnosis time, ART duration, viral load, immune parameters (e.g., CD4 count, CD8 count, CD4/CD8 ratio), first-line treatment regimen, and the proportion of change to the second-line treatment regimen were all matched between the three groups.

**Table 1:** Baseline characteristics

	High dose (*n* = 24)	Low dose (*n* = 24)	Control (*n* = 24)	*p* Value
Site [*n* (%)]				
Beijing	7 (29.17%)	6 (25.00%)	6 (25.00%)	0.931^b^
Yunnan	8 (33.33%)	7 (29.17%)	7 (29.17%)	0.937^b^
Xinjiang	6 (25.00%)	7 (29.17%)	8 (33.33%)	0.817^b^
Guangzhou	3 (12.50%)	4 (16.67%)	3 (12.50%)	0.890^b^
Age (years)	42.0 (33.3, 48.0)	42.5 (38.0, 45.8)	41.5 (35.5, 48.8)	0.959^a^
No. of male/female (*n*)	19/5	19/5	19/5	>0.999^b^
Weight (Kg)	59.0 (55.3, 65.0)	65.0 (55.3, 70.0)	61.0 (57.0, 63.9)	0.323^a^
BMI	20.80 ± 2.04	22.06 ± 2.04	21.40 ± 2.11	0.115^a^
Route of HIV infection [*n* (%)]				
Sexual	20 (83.33%)	16 (66.67%)	16 (66.67%)	0.330^b^
Blood/Injection drug use	2 (8.33%)	2 (8.33%)	1 (4.17%)	0.807^b^
Other or unknown	2 (8.33%)	6 (25.00%)	7 (29.17%)	0.171^b^
Diagnosis time (years)	4.13 (2.35, 5.23)	3.25 (2.92, 4.40)	3.58 (2.58, 5.71)	0.803^a^
ART time (years)	3.38 (2.12, 4.25)	3.13 (2.58, 4.06)	3.17 (2.29, 4.96)	0.739^a^
Viral load < 50 copies per mL (%)	100%	100%	100%	>0.999^b^
CD4 counts (cell/μL)	179 (144, 224)	181 (149, 200)	179 (161, 209)	0.935^a^
CD8 counts (cell/μL)	733 (476, 878)	558 (400, 868)	623 (466, 835)	0.509^a^
CD4/CD8 ratio	0.29 (0.17, 0.35)	0.31 (0.20, 0.41)	0.29 (0.20, 0.45)	0.639^a^
First-line drugs [*n* (%)]				
AZT + 3TC + NVP/EFV	13 (54.17%)	11 (45.83%)	11 (45.83%)	0.801^b^
D4T + 3TC + NVP/EFV	7 (29.17%)	6 (25.00%)	8 (33.33%)	0.817^b^
TDF + 3TC + NVP/EFV	2 (8.33%)	6 (25.00%)	3 (12.50%)	0.248^b^
Other	2 (8.33%)	1 (4.17%)	2 (8.33%)	0.807^b^

The data for age, weight, diagnosis time, ART time, CD4 counts, CD8 counts, CD4/CD8 ratio are the median (interquartile range [IQR]). The BMI is shown as the mean ± SE; other data are shown as *n* (%).

*3TC* lamivudine, *TDF* tenofovir, *D4T* stavudine, *EFV* efavirenz, *NVP* nevirapine.

^a^*P* value of continuous data between three groups: Kruskal–Wallis test or one-way ANOVA *t* test.

^b^*P* value of categorical data between three groups: Pearson *χ*^2^ test or Fisher’s exact test (two-tailed).

In addition, the pre-ART baseline median CD4 T cell counts [61 (21,155) vs. 53 (14,110) vs. 58 (39,170) cells/μL, *P* = 0.544], CD8 counts [532 (444,1067) vs. 658 (393,988) vs. 625 (288,1311) cells/μL, *P* = 0.847], and CD4/CD8 ratios [0.12 (0.09, 0.26) vs. 0.10 (0.03,0.17) vs. 0.11 (0.06, 0.22), *P* = 0.352] were also detected in the high-dose, low-dose, and control groups, respectively, and no significant difference was observed among the three groups (Fig. S1), which indicates that the patients possibly had the same immune status before ART.

**Fig. 2:**
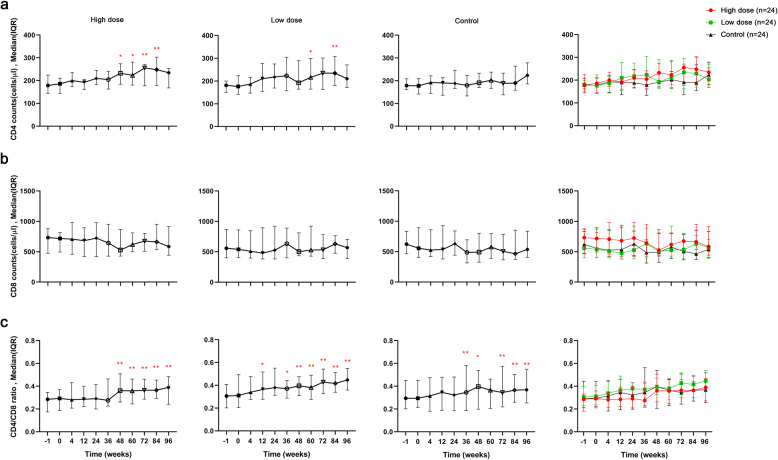
Longitudinal changes of peripheral CD4 counts, CD8 counts, and CD4/CD8 ratios according to treatment group. **a** CD4 counts; **b** CD8 counts; **c** CD4/CD8 ratios. *P < 0.05; **P < 0.01

The hUC-MSC groups had increased CD4 counts after 48 weeks of treatment (Fig. 2a). Further detailed study showed that the high-dose group had better CD4 T cell recovery at 48 weeks [232 (184,274), *P* = 0.038], 60 weeks [223 (180,281), *P* = 0.018], 72 weeks [256 (177,271), *P* < 0.001], and 84 weeks [248 (179,303), *P* < 0.001] compared with baseline [179 (144,224) cells/μL]. The low-dose group had better CD4 T cell recovery at 48 weeks [192 (164,290), *P* = 0.082], 60 weeks [216 (164,290), *P* = 0.023] and 84 weeks [229 (170,299), *P* = 0.002] compared with baseline [181 (149,200) cells/μL]. No such trend was observed for the control group (such as at 48 weeks [191 (170, 233), *P* = 1.000] and 96 weeks [224 (180, 279), *P* = 0.056] as compared with baseline [179 (161, 209) cells/μL]), and there was no statistical difference in CD4 T cell counts among the three groups (generalized estimating equations, *P* = 0.389).

Furthermore, we also calculated the changes in delta CD4 T cell counts between baseline and the different time points among the groups (Fig. S2a). The median delta CD4 counts increased significantly at 4 weeks [30 (−6, 47), *P* = 0.041], 36 weeks [26 (−11, 52), *P* = 0.038], 48 weeks [40 (3, 75), *P* < 0.001], 60 weeks [41 (9, 75), *P* = 0.031], 72 weeks [50 (24, 78), *P* < 0.001], 84 weeks [66 (17, 121), *P* < 0.001], and 96 weeks [41 (−8, 64), *P* < 0.001] compared with 0 weeks (0 cells/μL) in the high-dose group; at 12 weeks [28 (−20, 75), *P* = 0.021], 24 weeks [29 (−20, 75), *P* < 0.001], 36 weeks [41 (8, 104), *P* < 0.001], 48 weeks [37 (7, 103), *P* < 0.001], 60 weeks [50 (20, 88), *P* < 0.001], 72 weeks [55 (9, 110), *P* < 0.001], 84 weeks [60 (22, 110), *P* < 0.001], and 96 weeks [32 (7, 121), *P* < 0.001] compared with 0 weeks (0 cells/μL) in the low-dose group; and at 96 weeks [51 (13, 79), *P* < 0.001] compared with 0 weeks [0 (−5, 0) cells/μL] in the control group. However, there was no statistical difference in the changes in delta CD4 T cell counts among the three groups (generalized estimating equations, *P* = 0.228).

**Fig. 3:**
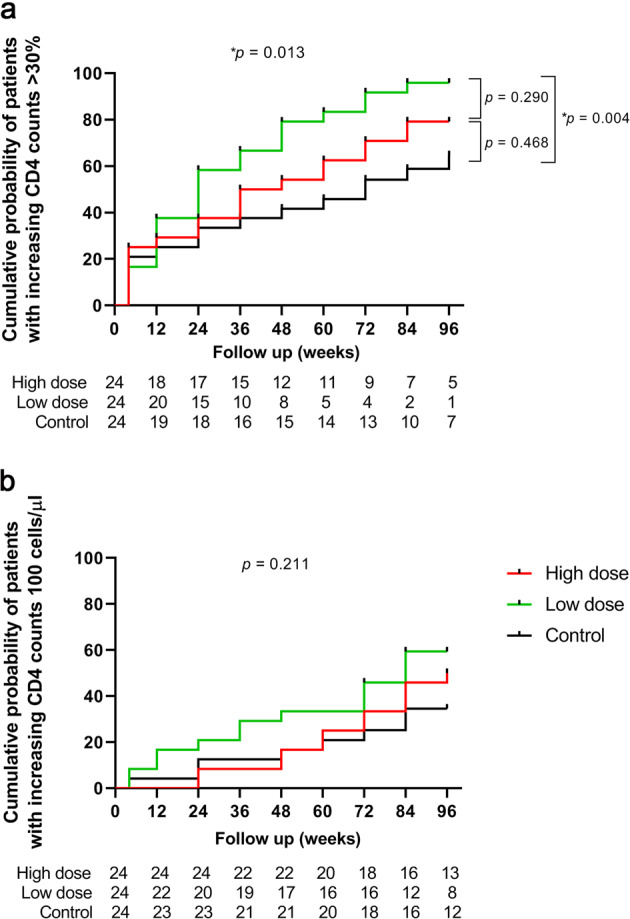
The cumulative probability of patients with an immunological response. Analyses of the cumulative probability of patients whose CD4 counts increased > 30% (**a**) or (**b**) >100 cells/μL compared with baseline

In addition, based on the concept of immunological responders, whose CD4 count increased >100 cells/μL or by 30% compared with baseline, we re-evaluated the treatment effect of hUC-MSCs, and observed a significant difference in the cumulative probability of patients with increased CD4 counts >30% between the three groups (Fig. 3a, log-rank test, *P* = 0.013). Further analysis showed that the low-dose group had significantly higher cumulative probability of patients with increased CD4 counts >30% than the control group (95.8% vs. 70.8%, *P* = 0.004), but no significant difference was observed between the low-dose vs. high-dose groups (*P* = 0.290) and between the high-dose group vs. the control group (*P* = 0.468). However, there was no statistical difference in the cumulative probability of patients with increased CD4 counts >100 cells/μL between the three groups (Fig. 3b, log-rank test, *P* = 0.211).

Compared with baseline, there was no statistical difference in CD8 counts within the high-dose, low-dose, and control groups (Fig. 2b). Moreover, CD8 counts between the three groups were not statistically different (generalized estimating equations, *P* = 0.436).

There was a significant difference for the CD4/CD8 ratio within all of three groups (Friedman test, all, *P* < 0.001). Further investigation found that, compared with baseline [high-dose group, 0.285 (0.174, 0.346); low-dose group, 0.306 (0.202, 0.408); control, 0.294 (0.201, 0.451)], the CD4/CD8 ratio increased significantly at 48 weeks [0.361 (0.261, 0.508), *P* = 0.002], 60 weeks [0.359 (0.245, 0.463), *P* = 0.001], 72 weeks [0.365 (0.287, 0.462), *P* < 0.001], 84 weeks [0.365 (0.293, 0.454), *P* < 0.001], and 96 weeks [0.390 (0.242, 0.484), *P* < 0.001] in the high-dose group; at 12 weeks [0.364 (0.276, 0.517), *P* = 0.035], 36 weeks [0.371 (0.287, 0.443), *P* = 0.048], 48 weeks [0.396 (0.309, 0.476), *P* = 0.003], 60 weeks [0.380 (0.275, 0.488), *P* = 0.001], 72 weeks [0.428 (0.327, 0.543), *P* < 0.001], 84 weeks [0.416 (0.339, 0.512), *P* < 0.001], and 96 weeks [0.447 (0.359, 0.548), *P* < 0.001] in the low-dose group; and at 36 weeks [0.346 (0.187, 0.582), *P* = 0.009], 48 weeks [0.397 (0.198, 0.538), *P* = 0.01], 72 weeks [0.345 (0.218, 0.575), *P* = 0.006], 82 weeks [0.365 (0.260, 0.503), *P* = 0.006], and 96 weeks [0.368 (0.253, 0.549), *P* < 0.001] in the control group (Fig. 2c). However, there was no significant statistical difference in the CD4/CD8 ratio between the three groups (generalized estimating equations, *P* = 0.078). Furthermore, we also calculated the changes in the delta CD4/CD8 ratio between baseline and the different time points among the groups (Fig. S2b). We observed different increasing trends of the delta CD4/CD8 ratios in each group as treatment time was prolonged, but observed no statistical difference in the changes in the delta CD4/CD8 ratios among the three groups (generalized estimating equations, *P* = 0.695).

There were no infusion-associated serious adverse events in any group. A total of 31 participants (43.6%) reported at least one adverse reaction within the 96-week follow-up after treatment: 12 (50.0%) in the high-dose group, 8 (33.3%) in the low-dose group, and 11 (45.8%) in the control group, but there was no significant difference between the three groups (*P* = 0.478). No grade 3 or 4 adverse events were reported. The most common adverse reactions, such as fever (self-limiting fever of 37–38 °C within 4 h after hUC-MSC transfusion), fatigue, and insomnia, were reported in six participants (25.0%) in the high-dose group, three participants (12.5%) in the low-dose group, and five participants (20.8%) in the control group (*P* = 0.538).

For other adverse reactions, such as cardiac disorders, gastrointestinal disorders, skin disorders, otolaryngologic disorders, respiratory system disorders, and hematological disorders, no significant differences were observed between the three groups (Table 2). In addition, cytokine release syndrome (CRS) was not observed in the high-dose group.

**Table 2:** Clinical adverse reactions within 96 weeks follow-up

	High dose (*n* = 24)	Low dose (*n* = 24)	Placebo (*n* = 24)	*P* value
Adverse reactions	12 (50.0%)	8 (33.3%)	11 (45.8%)	0.479
Skin disorders	1 (4.2%)	1 (4.2%)	2 (8.3%)	0.712
Hematological disorder	0	0	1 (4.2%)	0.363
Otolaryngology disorder	1 (4.2%)	1 (4.2%)	1 (4.2%)	>0.999
Respiratory system disorder	0	1 (4.2%)	1 (4.2%)	0.598
Cardiac disorders	2 (8.3%)	1 (4.2%)	0	0.352
Gastrointestinal disorders	2 (8.3%)	1 (4.2%)	0	0.352
Endocrine system disorders	0	0	0	–
Urogenital system disorders	0	0	0	–
Skeletal musculature disorders	0	0	1 (4.2%)	0.363
Neuropsychiatric disorders	0	0	0	–
Others (fever, fatigue, insomnia, and so on)	6 (25.0%)	3 (12.5%)	5 (20.8%)	0.538

Values are presented as *n* or *n* (%)

Data shown are the numbers of patients (%) experiencing adverse reactions from day 1 of the treatment to the 96-week follow-up checkpoints. All events were included in the categories shown

No HIV-1 load rebound, or significant increases in liver function enzymes, were observed throughout the study (Table S1). In addition, no opportunistic infections or AIDS-defining tumors were observed in the hUC-MSC-treated patients throughout the trial period. No antiretroviral regimen or compliance issues occurred during the trial. These observations indicate that the hUC-MSC transfusions were clinically and biologically well-tolerated in the patients.

## Discussion

This multicenter, double-blinded, randomized, placebo-controlled, dose-determination study was designed to prove the safety and treatment efficacy of hUC-MSC transfusion for INR patients with HIV-1, and to attempt to identify a well-tolerated dose of hUC-MSCs. We found that both doses of hUC-MSC (0.5 × 10^6^ and 1.5 × 10^6^ cells/kg body weight) were safe and well-tolerated, and no transfusion-related serious events occurred. Based on the present data, the absence of a significant difference in CD4 T cell counts and CD4/CD8 ratios among the three groups indicates that the hUC-MSC at both administered doses do not show significant effectiveness for immune recovery in our small cohort of INR HIV-infected patients. Therefore, a large cohort study is necessary to further investigate the therapeutic efficacy of hUC-MSC for INR HIV-infected patients in the future.

In spite of the successful treatment of ART in reducing HIV-related mortality and morbidity worldwide, INR patients still represent a special population with poor outcomes, with no obvious CD4 recovery following stable ART. Bone marrow hematopoiesis dysfunction, insufficient thymic output, residual virus replication, and aberrant immune activation all account for the immune reconstitution failure of INR patients. Different immune therapies such as IL-2 therapy have been used for increasing CD4 counts, but yield no clinical benefits due to the induced CD4 T cells lacking the naïve phenotype and having lower host defense capacity, so they have no effect on reducing the risk of opportunistic diseases,^16^ which means that both the quantity and quality of the renewed CD4 T cells are crucial. Previously, we conducted a prospective, open-label, randomized controlled clinical study that involved 13 INR patients.^14^ Seven patients in the treatment group received three i.v. allogenic UC-MSC (0.5 × 10^6^ cells/kg) transfusions on months 0, 1, and 2, while the six control patients received saline. After 12 months follow-up, the early and central memory CD4 T cells were increased, and T cell overactivation was decreased. It appears that MSC-based treatments may principally contribute to inhibiting the uncontrolled immune-inflammatory response, benefiting the recovery of functional CD4 T cells. Then, we redesigned the clinical trial (phase II) and enrolled entirely new patients who had not received phase I treatment of MSC as previously reported.^14^ The unanticipated finding in the present trial is that the increase in CD4+ T cells occurred at 48 weeks but not in the early phase after hUC-MSC transfusion, which has some consistency with our previous study in that the effect of MSCs was initiated 6 months after UC-MSC therapy was started. The above results indicate that hUC-MSCs may have a cumulative and delayed effect on CD4+ T cell recovery, but the reason is not known.

The CD4+ T cell count and CD4/8 ratio are two major indexes for evaluating immune reconstitution in HIV/AIDS patients.^17–20^ In a recent study, we found that CD4 ≥ 500 cells/μL together with CD4/CD8 ratio ≥ 0.8 were more accurate for evaluating immune restoration status.^21^ In the present study, there was no significant difference in CD4 and CD4/CD8 ratio recovery between the hUC-MSC treatment and control groups, but there were significant increasing trends of CD4+ T counts and CD4/CD8 ratios (Fig. 3a–c) and changes in delta CD4+ T counts and delta CD4/CD8 ratios (Fig. S2a, b) in the hUC-MSC transfusion patients after 48 weeks’ treatment. Here, the low number of patients achieving CD4/CD8 ratio normalization may have been caused by the lowest baseline CD4 T cell counts and CD4/CD8 ratios before ART (Fig. S1), so the importance of earlier ART should be emphasized. Although there was no statistical difference in the CD4 T cell counts and CD4/CD8 ratios among the three groups, we nevertheless observed the temporal recovery of the CD4+ T cells in the hUC-MSC transfusion group, which indicates that hUC-MSC treatment may contribute to immune reconstitution in INR patients. Therefore, the treatment effect of hUC-MSC transfusion for INR patients should be investigated in a larger sample.

Numerous previous studies have reported that the treatment effect of MSC transfusion shows significant dose dependence.^22^ It is critical to determine the most effective doses of hUC-MSCs before proceeding to large clinical trials and clinical application in the future. In the present study, we tripled the dose of hUC-MSCs to 1.5 × 10^6^ cells/kg body weight in the high-dose group, and expected to achieve a better treatment effect than with the low dose. However, to our surprise, the high-dose transfusion did not have the desired treatment effect. For the possible mechanisms of the lack of increased treatment effects for high-dose hUC-MSC transfusion, Saether et al. found that higher-dose hUC-MSC transfusion led to a significant decrease in anti-inflammatory M2 macrophages and promoted the secretion of pro-inflammatory cytokines from other cells, including IL-1β, IFN-γ, and IL-2, which may have led to the lack of added treatment effect as compared to the low-dose group.^23^ Furthermore, no significant difference was observed for the cumulative probability of patients achieving immunological response. Other groups have reported similar results, where increasing the dose of stem cells did not have the desired treatment effect,^24^ which suggests that different doses of MSCs might exert different treatment efficacy in a dose-independent manner.

Our study has its limitations. First, LOCF was the commonly used means of imputing data with dropouts. However, this method may induce bias in unpredictable ways. Second, we failed to explore the optimal repeated frequency and interval of administration. Compared to our phase I study, we had increased the number of injections and extended the treatment duration, but this was limited by the size of our experiment; the relationship between the number of injections, treatment interval, and therapeutic efficacy was not examined. Third, there is a new understanding about the *P*-value in statistics. A *P*-value of <0.05 is sometimes interpreted as meaning that there is a stronger relationship between two variables. However, statistical significance does not mean that the null hypothesis is true. In the present study, some of our conclusions were based solely on a *P*-value, which may not mean that they are actually clinically significant. Finally, the number of patients enrolled in our study remains insufficient. Therefore, a larger sample is needed to confirm the treatment effect of hUC-MSC transfusion in INR patients until the completion of phase III clinical trial. Our team will initiate this phase III clinical trial, the investigator and each team member have relevant qualifications and a wealth of experience for this project. A nationwide network of multi-hospital partnerships has been established, which will provide adequate patients and multi-center requirements for the phase III clinical trial. Currently, the project is directly supported by the National Science and Technology Major Project of the Ministry of Science and Technology of China, and more funds will be raised to support the project in the future. Therefore, it appears feasible for our team to conduct a phase III effectiveness trial.

In conclusion, we report that both doses of hUC-MSC transfusion were well-tolerated for treating INR patients with HIV-1. The low-dose outperformed the high-dose transfusion, and at least in part, improved host immune reconstitution in the patients, which presents a promising novel therapeutic approach for such patients in combination with ART. In the future, a phase III clinical trial and relevant mechanistic investigation are needed to evaluate the efficacy of hUC-MSC treatment in INR HIV-infected patients.

## Materials and methods

### Study design and participants

We performed this phase II, multicenter, randomized, double-blinded, placebo-controlled, dose determination (0.5 and 1.5 x 10^6^ cells/kg) safety trial (ClinicalTrials.gov Identifier: NCT01213186) of intravenous (i.v.) allogeneic hUC-MSCs for immune reconstitution in INR patients with HIV-1 infection. Institutional review board approvals for the treatment protocol were obtained from all centers involved before the initiation of patient enrollment: Fifth Medical Center of Chinese PLA General Hospital, Beijing, China; Yunnan Provincial Hospital of Infectious Diseases, Kunming, China; Xinjiang Uygur Autonomous Regional the Sixth People’s Hospital, Urumqi, China; Guangzhou Eighth People’s Hospital, Guangzhou Medical University, Guangzhou, China. The eligible patients enrolled in this study must have received ART for at least 24 months with full viral suppression (plasma HIV RNA load <50 copies/mL), and simultaneously their CD4 counts should be >50 cells/μL and <250 cells/μL within 6 months before enrollment. The exclusion criteria were: history of autoimmune disease; any malignancy, opportunistic infections, and AIDS-defining tumors; pregnancy; concomitant or previous treatment with interferons; anti-HIV vaccines, steroids, or any other immunomodulators within the previous 12 months. Each patient agreed to and signed an institutional review board-approved statement of informed consent.

### The hUC-MSC transfusions

The hUC-MSCs were prepared according to our previously described protocols.^14^ In brief, the umbilical cord vessels were removed, and the mesenchymal tissue in Wharton’s jelly was diced into cubes, washed, and finally seeded into a tissue culture flask. After 12–15-day culture, the remnants of the cord fragments were removed, and the adherent cells were cultured, generated, and collected between the third and fourth passages. The collected hUC-MSCs were resuspended and transfused i.v. into the patients at 0.5 × 10^6^ cells/kg body weight (low dose) or 1.5 × 10^6^ cells/kg body weight (high dose), respectively. Before use in transfusions, the hUC-MSCs underwent quality control testing, including evaluation of phenotypes, cytokine-producing profiles, and the capacity for osteogenesis and adipogenesis (Fig. S3). In addition, the hUC-MSCs were tested for pathogens at every passage and prior to injection. All patients received a single i.v. infusion of hUC-MSCs or placebo suspension delivered at a rate of 2 mL/min. The i.v. tubing was coated with an opaque wrap to obscure any identifying features of the infusion.

### Randomization and masking

The patients were enrolled by the study investigators. Randomization was performed by a centralized randomization system based on a randomization list generated by an independent statistician using SAS software. The patients were randomly assigned on a ratio of 1:1:1 to 0.5 × 10^6^ hUC-MSC/kg body weight, 1.5 × 10^6^ hUC-MSC/kg body weight, or placebo (saline) groups. The participants, investigators, study site personnel, and funding institution staff were masked to treatment assignment throughout the 96-week study period.

### Procedures

hUC-MSC or saline transfusions were administered six times to each patient on day 0, and at 4 weeks, 12 weeks, 24 weeks, 36 weeks, and 48 weeks (treatment period). The patients attended follow-up checkups for a total of 24 months from the beginning of the study. During the treatment and follow-up period, all patients continued to receive ART (unchanged regimen for each patient after enrollment in this clinical trial) (Fig. 1b). Patients who did not attend the last follow-up check points (96 weeks) were regarded as lost to follow-up. The clinical safety was assessed via an interim medical history and physical examination through the reporting of adverse reactions, such as skin disorders, otolaryngology disorder, cardiac disorders, gastrointestinal disorder, and other disorders (e.g., fever, fatigue, insomnia), as well as the occurrence of opportunistic infections and tumors. All patients underwent regular examination for liver function, kidney function, routine blood tests, coagulation markers, and virological and immunological parameters.

### Endpoint

The primary endpoint was to evaluate the changes in CD4 counts as compared with baseline. We also calculated the cumulative probability of immunological responders, which was defined as CD4 counts increased by >100 cells/µL or by 30% as compared with baseline within one year of treatment.^25,26^ The secondary endpoint was to measure the changes in CD8 counts and the CD4/CD8 ratio, the number of participants with adverse reaction, quality of life, and the occurrence rate of tumor and opportunistic infections.

### Statistical analysis

The sample size was calculated using PASS software (version 11.0). According to our previous phase I pilot study, we assumed that a >50% increase in CD4 count could be observed in 85% of patients in the treatment group and in 45% of patients in the placebo group. A sample size of 19 patients per group achieved at least 80% power for detecting a difference between the groups. Considering a dropout rate of 20%, we decided on a total sample size of 72, with 24 participants per group.

The demographic and baseline characteristics were summarized for all randomized patients. The safety analyses were applied to all randomized patients and at least one treatment received with the observation data available in the study. In the case of missing data, the last evaluable assessment was carried forward to the endpoint (LOCF). Categorical data were analyzed using the *χ*^2^ test or Fisher’s exact test; multiple *χ*^2^ comparisons were performed based on a Bonferroni-adjusted α value of 0.017 when relevant. If required by the condition, continuous data were analyzed using the Kruskal–Wallis test, Friedman test, analysis of variance (ANOVA), or repeated measurement test, and the Nemenyi test or least significant difference (LSD) test for multiple comparisons between groups was used if there was a significant difference across treatment groups. The effect of the intervention was evaluated using generalized estimating equations.

We estimated the cumulative probabilities of immunological responders (CD4 T cell counts >100 cells/μL or increased by 30%) using Kaplan–Meier analysis stratified by the different treatment regimens, and determined statistical significance using the log-rank test for running parallel hazard rates and a two-stage procedure for crossing hazard rates. All significance tests performed were two-sided. *P*-values < 0.05 were deemed statistically significant. All analyses were conducted using SAS software version 9.4 and GraphPad Prism 8.0.

## Supplementary information

Supplemental Material

## Data Availability

All of the data generated and analyzed during this study are included in our manuscript.
