# Supplementary material for: Human umbilical cord mesenchymal stem cell transfusion in immune non-responders with AIDS: a multicenter randomized controlled trial
Source: Signal Transduct Target Ther. 2021 Jun 9;6:217. doi: 10.1038/s41392-021-00607-2 (PMC8187429; doi:10.1038/s41392-021-00607-2)
Supplement: Supplementary file 1 — Supplemental Material [file 41392_2021_607_MOESM1_ESM.pdf]

Supplementary information for

Human umbilical cord mesenchymal stem cell transfusion in immune non-responders with AIDS: A multicenter randomized controlled trial

Lifeng Wang[a, #], Zheng Zhang[b, #], Ruonan Xu[a,#], Xicheng Wang[c, #], Zhanjun Shu[d], Xiejie Chen[e], Siyu Wang[a], Jiaye Liu[a], Yuanyuan Li[a], Li Wang[c], Mi Zhang[c], Wei Yang[d], Ying Wang[d], Huihuang Huang[a], Bo Tu[a], Zhiwei Liang[e], Linghua Li[e], Jingxin Li[f], Yuying Hou[a], Ming Shi[a], Fu-Sheng Wang[a,*]

# Those authors contributed equally to this work

Correspondence to Fu-Sheng Wang, PhD, MD,

Treatment and Research Center for Infectious Diseases, Fifth Medical Center of Chinese PLA General Hospital, Beijing, China. Tel: +86 10 66933332; Fax: +86 10 63879735; fswang302@163.com

Supporting Results

Fig. S1. The pre-ART baseline CD4/CD8+ T cell counts and CD4/CD8 ratios among the three groups.

Fig. S2. The changes in delta CD4+ T cell counts and CD4/CD8 ratios between baseline and the different time points among the three groups.

Fig. S3. MSC assessment.

Table S1. Safety and tolerability of hUC-MSC transfusions in HIV-1–infected patients

Supporting Results

Fig. S1. The pre-ART baseline CD4/CD8+ T cell counts and CD4/CD8 ratios among the three groups. (a) CD4+ T cell counts, (b) CD8+ T cell counts, (c) CD4/CD8 ratios. In some of the patients, only the pre-ART CD4+ T count was detected, but not the pre-ART CD8+ T cell count. Accordingly, there were 13 patients in the high-dose group, 14 patients in the low-dose group, and 13 patients in the control group in (b) and (c).

Fig. S2. The changes in delta CD4+ T cell counts and CD4/CD8 ratios between baseline and the different time points among the three groups. (a) Delta CD4 counts, (b) delta CD4/CD8 ratios. *$P < 0.05$; **$P < 0.01$.

Fig. S3. MSC assessment. Cell surface marker of UC-MSCs at P4 were positive for CD73, CD90, and CD105, and were negative for CD19, CD11b, CD34, CD45, and HLA-DR.

Table S1. Safety and tolerability of hUC-MSC transfusions in HIV-1–infected patients

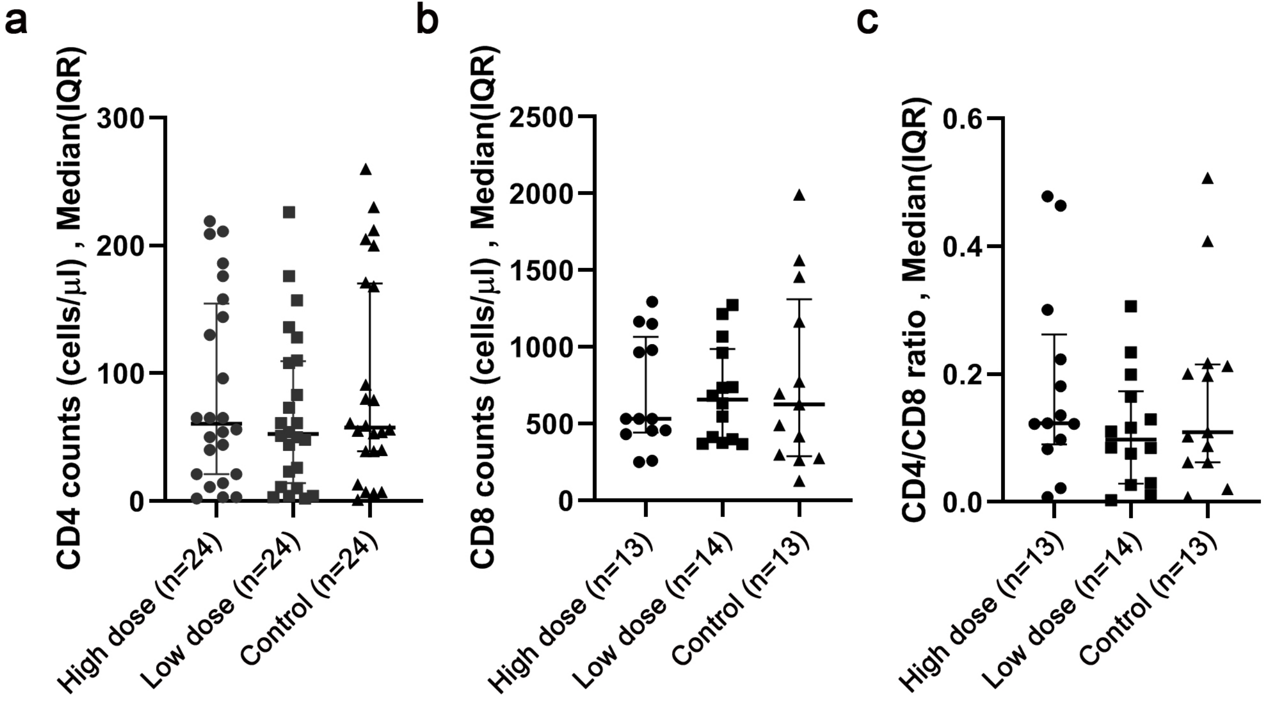
a
b
c
CD4 counts (cells/μl) , Median(IQR)
300
200
100
0
High dose (n=24)
Low dose (n=24)
Control (n=24)
CD8 counts (cells/μl) , Median(IQR)
2500
2000
1500
1000
500
0
High dose (n=13)
Low dose (n=14)
Control (n=13)
CD4/CD8 ratio , Median(IQR)
0.6
0.4
0.2
0.0
High dose (n=13)
Low dose (n=14)
Control (n=13)

Fig S1

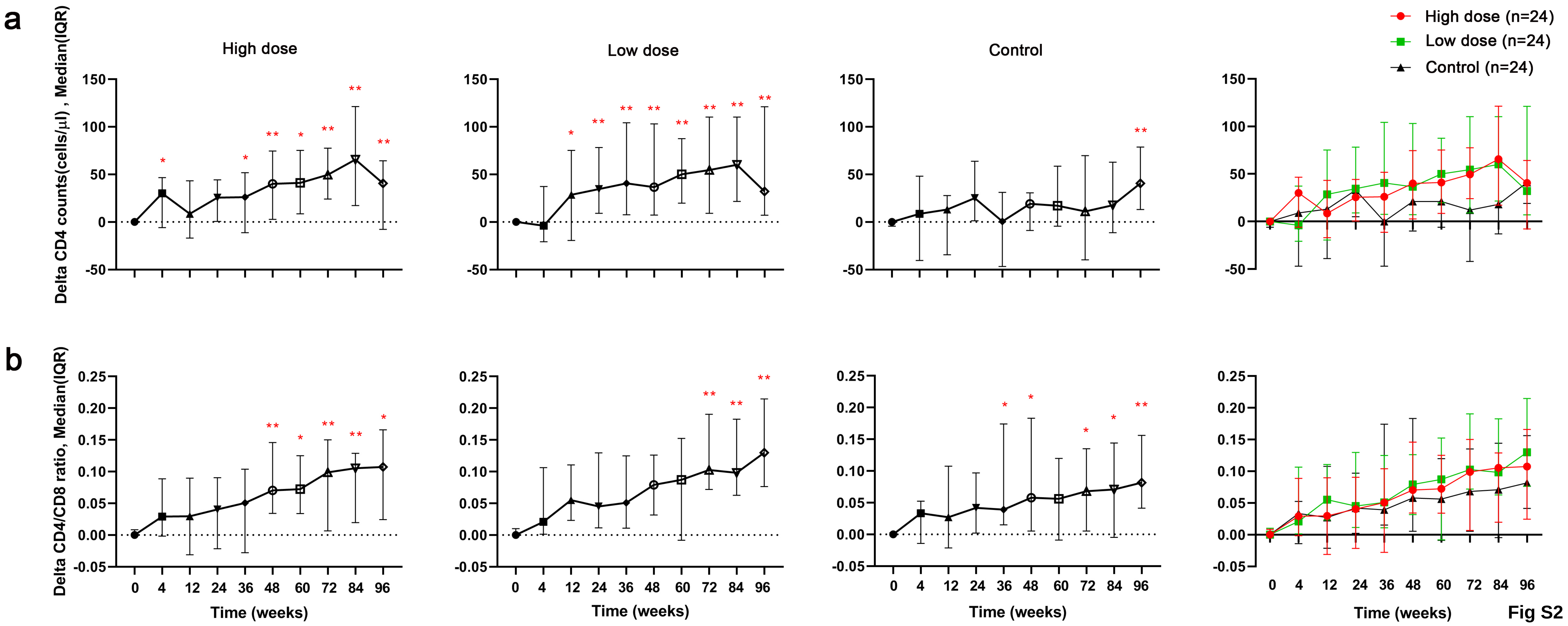
a
b
High dose
Low dose
Control
High dose (n=24)
Low dose (n=24)
Control (n=24)
Delta CD4 counts(cells/μl) , Median(IQR)
Delta CD4/CD8 ratio, Median(IQR)
Time (weeks)
150
100
50
0
-50
0.25
0.20
0.15
0.10
0.05
0.00
-0.05
0 4 12 24 36 48 60 72 84 96

Fig S2

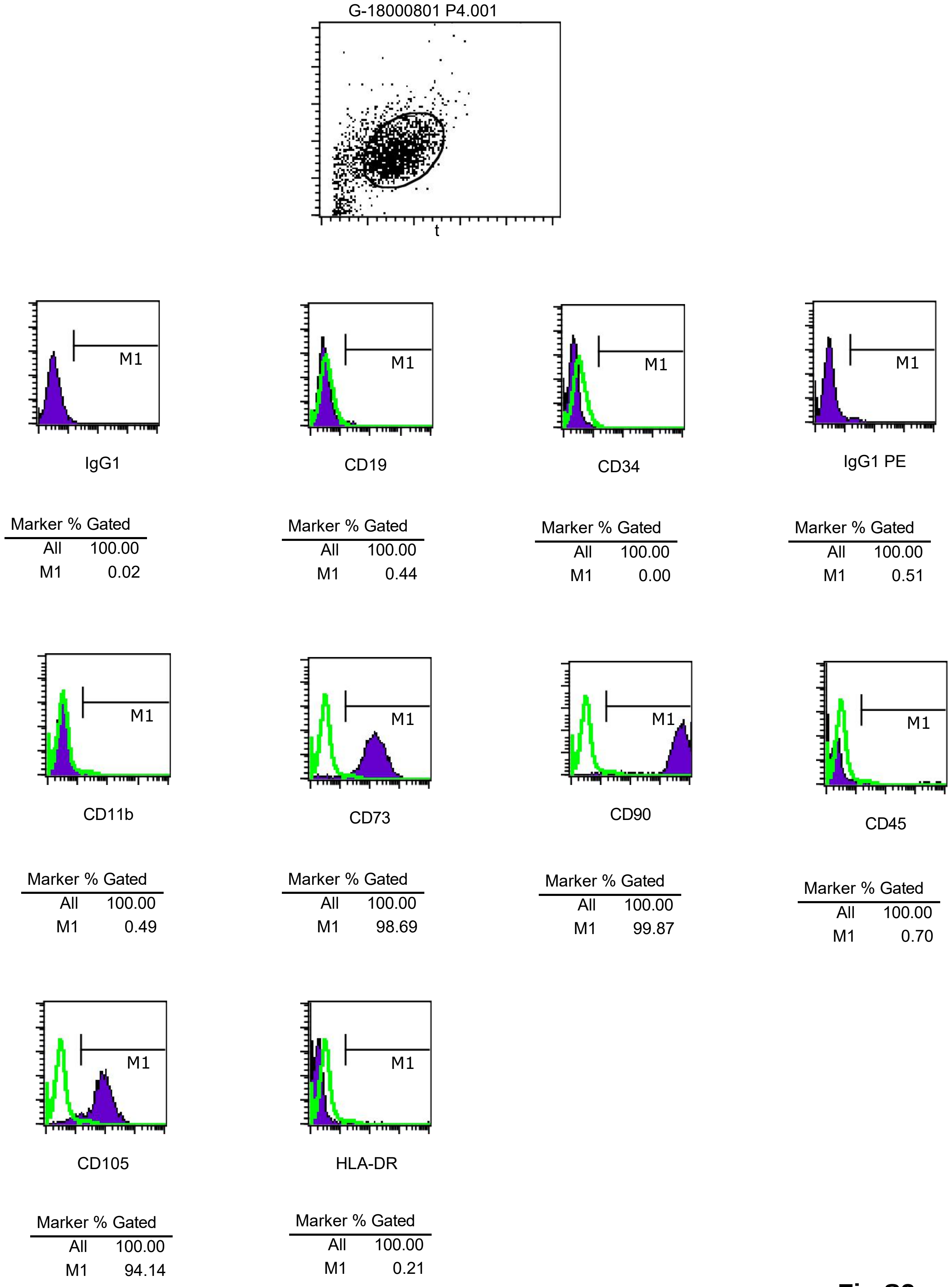

G-18000801 P4.001
t
M1
IgG1
Marker % Gated
All 100.00
M1 0.02
CD19
Marker % Gated
All 100.00
M1 0.44
CD34
Marker % Gated
All 100.00
M1 0.00
IgG1 PE
Marker % Gated
All 100.00
M1 0.51
CD11b
Marker % Gated
All 100.00
M1 0.49
CD73
Marker % Gated
All 100.00
M1 98.69
CD90
Marker % Gated
All 100.00
M1 99.87
CD45
Marker % Gated
All 100.00
M1 0.70
CD105
Marker % Gated
All 100.00
M1 94.14
HLA-DR
Marker % Gated
All 100.00
M1 0.21

Fig S3

Table S1. Safety and tolerability of hUC-MSC transfusions in HIV-1-infected patients

	Groups	-1 weeks	12 Weeks	24 Weeks	36 Weeks	48 Weeks	72 Weeks	96 Weeks
**WBC** (10^9/L)	High	4.00 (3.44,5.47)	4.22 (3.43,5.30)	4.58 (3.49,5.10)	4.76 (3.84,5.41)	4.83 (3.64,5.34)	4.47 (3.60,5.01)	4.70 (4.03,5.69)
	Low	4.56 (3.92,5.15)	4.91 (3.79,6.20)	5.03 (3.89,6.34)	5.03 (4.01,6.19)	4.63 (3.81,5.25)	4.78 (4.03,6.32)	5.38 (4.22,5.80)
	Control	4.38 (3.71,6.45)	4.43 (3.65,5.64)	4.75 (3.83,5.60)	4.18 (3.47,5.24)	4.67 (3.93,6.18)	5.23 (3.37,6.24)	4.60 (4.29,6.34)
**Neutrophil**	High	2.35 (1.91,3.49)	2.65 (2.05,3.21)	2.60 (2.10,3.47)	2.77 (2.06,3.55)	2.77 (2.14,3.38)	2.45 (2.17,3.15)	3.00 (2.35,3.79)
(10^9/L)	Low	2.74 (2.49,3.48)	3.09 (2.23,4.29)	3.24 (2.54,4.25)	3.36 (2.32,4.04)	2.95 (2.35,3.23)	2.80 (2.24,3.41)	3.03 (2.42,4.65)
	Control	3.35 (2.44,5.08)	2.89 (2.00,3.46)	2.73 (2.23,3.40)	2.59 (1.77,3.26)	2.83 (2.17,4.57)	3.14 (2.00,3.81)	3.09 (1.99,3.98)
**Monocyte**	High	0.26 (0.17,0.37)	0.27 (0.19,0.32)	0.24 (0.17,0.35)	0.26 (0.21,0.41)	0.32 (0.24,0.41)	0.24 (0.18,0.33)	0.33 (0.21,0.39)
(10^9/L)	Low	0.31 (0.23,0.37)	0.33 (0.23,0.41)	0.29 (0.23,0.43)	0.28 (0.21,0.34)	0.29 (0.21,0.37)	0.29 (0.22,0.38)	0.36 (0.28,0.43)
	Control	0.31 (0.22,0.42)	0.29 (0.23,0.42)	0.30 (0.25,0.38)	0.29 (0.24,0.40)	0.33 (0.24,0.43)	0.34 (0.23,0.46)	0.33 (0.23,0.49)
**Lymphocyte**	High	1.33 (0.93,1.51)	1.19 (0.95,1.60)	1.29 (0.93,1.57)	1.31 (0.96,1.63)	1.18 (0.98,1.42)	1.42 (1.03,1.66)	1.30 (1.09,1.57)
(10^9/L)	Low	1.22 (0.94,1.53)	1.27 (1.01,1.72)	1.24 (0.98,1.64)	1.23 (1.03,1.69)	1.31 (0.91,1.71)	1.49 (0.99,1.70)	1.39 (1.02,1.72)
	Control	1.36 (1.04,1.48)	1.25 (1.02,1,62)	1.36 (1.03,1.57)	1.19 (0.95,1.51)	1.21 (1.00,1.66)	1.25 (0.92,1.69)	1.36 (1.09,1.69)
**RBC**	High	4.31 (3.75,4.86)	4.11 (3.58,4.92)	4.21 (3.75,4.92)	4.24 (3.64,4.94)	4.06 (3.72,5.01)	4.25 (4.01,5.02)	4.11 (3.64,4.90)
(10^{12}/L)	Low	3.97 (3.53,4.34)	4.00 (3.63,4.39)	4.10 (3.55,4.55)	3.97 (3.61,4.38)	4.16 (3.80,4.46)	4.26(3.75,4.73)	4.19 (3.81,4.68)
	Control	3.99 (3.53,4.48)	4.01 (3.51,4.38)	4.09 (3.49,4.39)	3.80 (3.38,4.41)	3.90 (3.48,4.47)	3.94 (3.55,4.34)	4.07 (3.44,4.42)
**Platelet**	High	202.0 (130.3,237.8)	202.5 (143.5,227.3)	183.0 (147.8,224.3)	208.5 (149.8,242.5)	212.0 (144.0,233.0)	211.0 (141.0,241.0)	182.0 (164.0,264.0)
(10^9/L)	Low	178.0 (150.0,216.5)	167.0 (138.5,213.3)	176.0 (132.0,206.5)	179.0 (143.0,223.5)	190.0 (155.0,220.0)	187.5 (158.3,219.0)	187.0 (152.0,226.0)
	Control	192.0 (163.5,229.0)	194.5 (156.3,235.0)	189.0 (163.8,223.0)	192.0 (164.5,229.3)	198.0 (158.3,241.3)	194.0 (163.0,265.5)	202.5 (165.5,255.0)
**Hemoglobin**	High	150.5 (136.0,163.5)	144.5 (131.3,163.5)	154.5 (131.3,163.5)	156.5 (140.3,166.5)	152.0 (135.5,161.5)	152.0 (142.0,166.0)	154.0 (135.0,164.0)
(g/L)	Low	143.5 (129.3,154.8)	146.0 (130.3,157.0)	146.0 (137.5,159.8)	148.0 (134.3,155.0)	143.0 (131.0,157.0)	150.0 (132.3,161.8)	153.0 (139.0,164.0)
	Control	144.5 (139.3,157.5)	146.5 (138.3,159.8)	146.0 (138.3,158.8)	142.5 (133.3,146.8)	142.5 (127.8,155.5)	146.0 (132.0,150.5)	144.5 (133.8,158.3)
**ALB** (g/L)	High	46.0 (44.0,48.0)	47.0 (43.3,49.5)	47.0 (44.0,52.0)	47.0 (43.8,50.1)	48.6 (45.0,52.1)	47.0 (43.9,50.4)	46.0 (44.6,48.8)
	Low	45.4 (44.1,49.9)	45.5 (42.5,49.8)	45.9 (44.1,49.6)	47.1 (44.0,49.2)	46.6 (45.0,48.0)	44.9 (42.9,51.0)	47.0 (43.8,49.5)
	Control	46.5 (43.9,49.9)	46.4 (45.1,49.1)	47.0 (43.2,50.1)	46.2 (44.0,49.2)	45.2 (43.0,49.0)	46.0 (42.1,48.5)	45.0 (44.0,48.2)
**ALT** (U/L)	High	20.0 (16.3,32.0)	25.5 (18.5,38.0)	25.5 (17.3,49.8)	27.5 (19.0,35.5)	21.0 (17.0,34.0)	24.0 (18.0,33.0)	23.0 (20.0,35.0)
	Low	25.0 (18.3,43.5)	26.0 (17.0,37.3)	26.0 (20.3,42.8)	23.5 (19.3,39.0)	27.5 (18.5,41.0)	29.5 (15.3,37.8)	27.0 (21.0,39.0)
	Control	25.5 (15.5,33.5)	22.5 (13.3,33.0)	22.5 (12.3,37.3)	24.0 (15.3,30.3)	20.0 (14.0,31.2)	23.0 (14.0,31.0)	21.5 (13.0,25.3)
**AST** (U/L)	High	21.5 (18.3,26.5)	22.0 (18.3,26.5)	23.0 (19.0,25.3)	21.0 (16.5,28.0)	21.0 (17.0,25.0)	26.0 (19.0,28.0)	23.0 (21.0,29.0)
	Low	24.5 (14.5,35.8)	25.0 (17.0,32.3)	21.0 (15.3,30.5)	21.5 (16.0,29.8)	22.5 (16.5,33.5)	21.5 (18.5,28.5)	22.0 (18.0,33.0)
	Control	19.5 (16.3,27.3)	20.5 (18.3,25.8)	20.0 (16.3,29.8)	20.5 (15.3,25.5)	20.5 (16.0,25.8)	19.0 (17.0,25.5)	21.0 (16.0,23.3)
**TBIL**	High	8.2 (6.3,11.1)	8.2 (5.1,12.1)	8.0 (4.8,12.3)	7.9 (6.1,13.6)	8.6 (5.5,13.3)	9.1 (6.1,13.8)	10.2 (6.2,18.9)
(μmol/L)	Low	9.4 (7.6,12.9)	8.5 (5.9,13.3)	7.4 (6.3,12.5)	8.6 (6.5,14.7)	8.8 (7.0,13.2)	9.4 (7.3,13.4)	7.8 (5.9,18.4)
	Control	8.5 (6.2,9.9)	8.9 (6.4,11.8)	7.7 (6.4,9.5)	7.7 (5.5,11.1)	8.0 (5.4,10.6)	7.6 (5.8,9.2)	8.6 (6.3,10.2)
**BUN**	High	4.7 (3.8,5.6)	5.0 (4.1,6.4)	4.6 (4.1,5.5)	5.0 (3.9,5.9)	4.7 (4.0,5.7)	4.8 (3.5,5.6)	5.0 (4.4,5.7)
(mmol/L)	Low	4.7 (3.5,5.8)	5.0 (3.9,5.8)	5.0 (3.9,5.5)	5.1 (3.8,6.8)	4.7 (3.2,6.1)	4.7 (3.8,5.6)	5.0 (3.5,6.2)
	Control	4.5 (3.5,5.5)	4.1 (3.4,5.4)	4.5 (3.5,5.3)	4.4 (3.4,5.5)	4.4 (3.5,6.0)	4.2 (3.9,5.2)	5.2 (3.9,6.0)
**Cr** (μmol/L)	High	76.0 (68.3,86.0)	75.0 (65.0,86.5)	74.5 (64.3,82.8)	75.0 (70.0,79.0)	77.0 (72.0,83.0)	70.0 (62.0,81.0)	72.0 (65.3,79.5)
	Low	69.5 (55.5,83.5)	74.0 (59.8,84.5)	74.5 (65.3,88.0)	76.0 (55.0,82.0)	72.5 (54.5,80.5)	70.5 (53.0,88.3)	72.0 (61.5,83.5)
	Control	69.5 (60.0,76.8)	72.5 (60.8,84.0)	65.5 (58.8,82.5)	69.0 (61.3,80.0)	66.0 (59.0,81.5)	66.0 (58.5,77.0)	65.0 (61.5,72.0)
**PT** (s) #	High	11.6 (10.6,13.3)	-	-	-	10.8 (10.3,12.2)	-	11.0 (10.0,11.3)
	Low	12.0 (10.8,13.1)	-	-	-	11.7 (10.3,12.6)	-	10.8 (10.4,12.8)
	Control	11.5 (10.8,13.1)	-	-	-	11.3 (10.7,12.1)	-	11.1 (10.3,11.8)
**PA** (%) #	High	92.0 (83.0,101.2)	-	-	-	96.3 (81.2,103.2)	-	112.0 (102.5,117.8)
	Low	100.0 (86.1,107.0)	-	-	-	94.5 (74.7,106.1)	-	117.1 (97.0,123.5)
	Control	92.8 (86.6,104.2)	-	-	-	94.9 (83.9,100.0)	-	102.2 (79.3,123.7)

WBC, White blood cell; RBC, Red blood cell; ALB, albumin; ALT, Alanine transaminase; AST, Aspartate Transaminase; TBIL, Total bilirubin; BUN, blood urea nitrogen; Cr, creatinine; PT, Prothrombin time; PA, Prothrombin activity. High: high-does hUC-MSC transfusion group; Low: low-dose hUC-MSC transfusion group; Control: Placebo group

#, the data were only collected at -1 week, 48 weeks, and 96 weeks as designed in our clinical trial.
